# Supplementary material for: Clinical Validation of a Colorimetric Loop-Mediated Isothermal Amplification Using a Portable Device for the Rapid Detection of SARS-CoV-2
Source: Diagnostics (Basel). 2023 Apr 6;13(7):1355. doi: 10.3390/diagnostics13071355 (PMC10093461; doi:10.3390/diagnostics13071355)
Supplement: Supplementary file 1 [file diagnostics-13-01355-s001.zip › Supplementary information-MDPI.pdf]

Supplementary information

Table S1. Sequences of gene E and N of SARS-CoV-2 used as positive controls.

Gene E target sequence	Gene N target sequence
TGAGTACGAACTTATGTACTCATTCGTTT<br>CGGAAGAGACAGGTACGTTAATAGTTAA<br>TAGCGTACTTCTTTTTCTTGCTTTCGTGGT<br>ATTCTTGCTAGTTACACTAGCCATCCTTA<br>CTGCGCTTCGATTGTGTGCGTACTGCTGC<br>AATATTGTTAACGTGAGTCTTGTAAAACC<br>TTCTTTTTACGTTTACTCTCGTGTTAAAAA<br>TCTGAA	ACCAGGAACTAATCAGACAAGGAACT<br>GATTACAAACATTGGCCGCAAATTGC<br>ACAATTTGCCCCCAGCGCTTCAGCGTT<br>CTTCGGAATGTCGCGCATTGGCATGGA<br>AGTCACACCTTCGGGAACGTGGTTGAC<br>CTACACAGGTGCCATCAAATTGGATG<br>ACAAAGATCCAAATTTCAAAGATCAA<br>GTC

Table S2. LAMP primer sequences.

Gene E	Sequence
E1-F3	TGAGTACGAACTTATGTACTCAT
E1-B3	TTCAGATTTTTAACACGAGAGT
E1-FIP	ACCACGAAAGCAAGAAAAAGAAGTTCGTTTCGGAAGAGACAG
E1-BIP	TTGCTAGTTACACTAGCCATCCTTAGGTTTTACAAGACTCACGT
E1-LF	CGCTATTAACTATTAACG
E1-LB	GCGCTTCGATTGTGTGCGT
**Gene N**	**Sequence**
N2-F3	ACCAGGAACTAATCAGACAAG
N2-B3	GACTTGATCTTTGAAATTTGGATCT
N2-FIP	TTCCGAAGAACGCTGAAGCGGAACTGATTACAAACATTGGCC

N2-BIP	CGCATTGGCATGGAAGTCACAATTTGATGGCACCTGTGTA
N2-LF	GGGGGCAAATTGTGCAATTTG
N2-LB	CTTCGGGAACGTGGTTGACC

**Hs_rActin**	**Sequence**
ACT-F3	AGTACCCCATCGAGCACG
ACT-B3	AGCCTGGATAGCAACGTACA
ACT-FIP	GAGCCACACGCAGCTCATTGTATCACCAACTGGGACGACA
ACT-BIP	CTGAACCCCAAGGCCAACCGGCTGGGGTGTTGAAGGTC
ACT-LF	TGTGGTGCCAGATTTTCTCCA
ACT-LB	CGAGAAGATGACCCAGATCATGT

Table S3. Reproducibility data. The reproducibility test was performed with a serial dilution of each positive control (pUC17_N and pUC17_E) at a final concentration of 10^3 (level 1), 10^4 (level 2) and 10^5 (level 3) copies per μL, each.

**Run data**		**Level 1 - 10^3**	**Level 2 - 10^4**	**Level 3 - 10^5**
Operator and date	Positive results	Replicates	Replicates	Replicates
Operator 1 - Dec 10, 2020<br>Operator 2 - Dec 11, 2020<br>Operator 3 - Dec 14, 2020	100%	10/10	10/10	10/10
Operator 1 - Dec 10, 2020<br>Operator 2 - Dec 11, 2020<br>Operator 3 - Dec 14, 2020	100%	10/10	10/10	10/10

Operator 1 - Dec 10, 2020<br>Operator 2 - Dec 11, 2020<br>Operator 3 - Dec 14, 2020	100%	10/10	10/10	10/10		

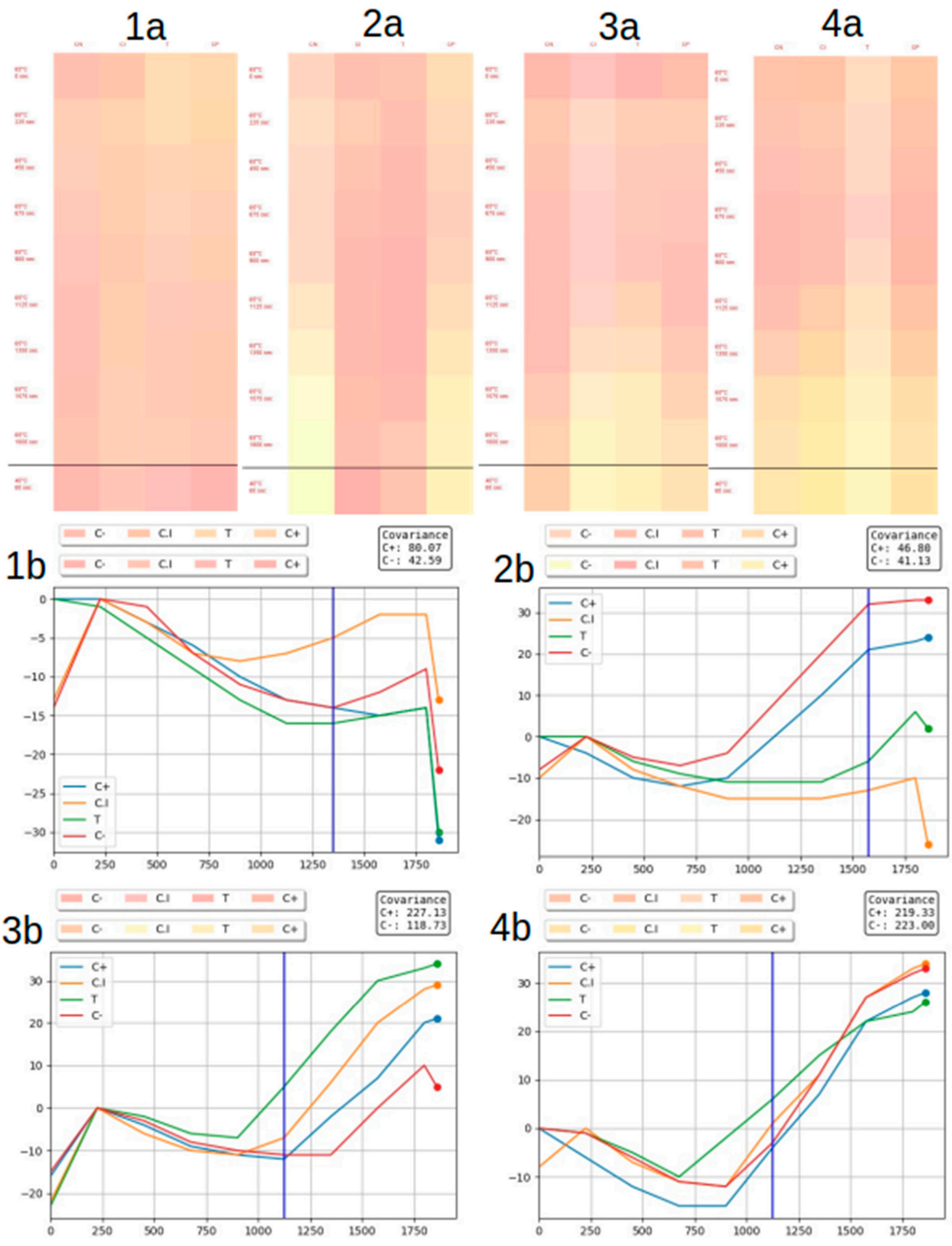

Figure S1. Software images obtained from the Limit of Detection (LoD) test. Figure 1a-b represents the non-template control. Figure 2a-b represents the 2.44×10^2 and 1.22×10^2 (left to right) genomic copies per reaction. Figure 3-ab represents the 9.77×10^2 and 4.88×10^2 (left to right) genomic copies per reaction. Figure 4a-b represents the 1.95×10^3 genomic copies per reaction.

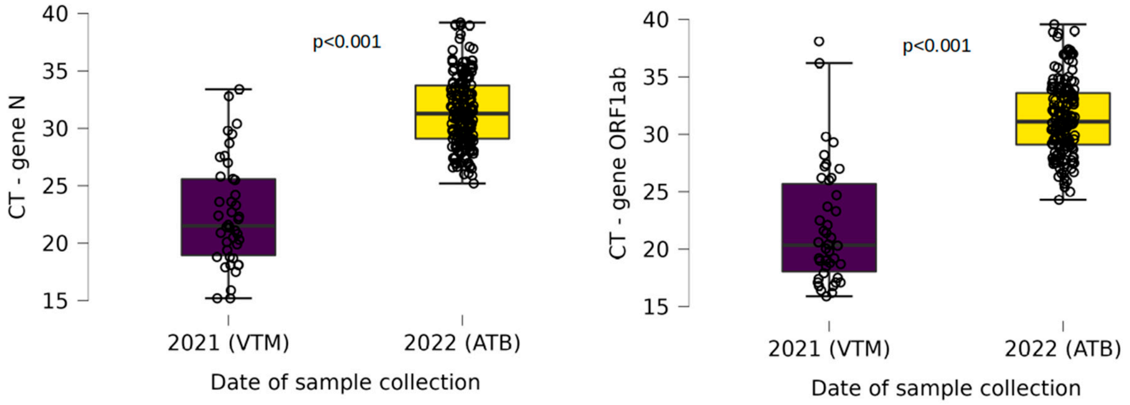

Figure S2. RT-qPCR CTs for samples collected in viral transport media (VTM) or antigen-test buffer (ATB). CTs for the N gene are shown in the left image, and the ORF1ab gene is in the right image. For both genes, we saw a statistically significant difference between sample solutions (p < 0.001).

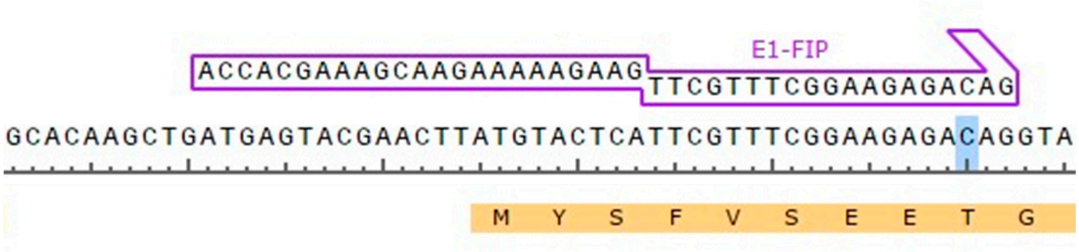

Figure S3. Omicron variant mutation at E gene (T9I). In purple is the sequence of our FIP primer targeting the E gene, demonstrating that the mutation of the Omicron variant affected the primer annealing.
